# Conservative Management of Tourette Syndrome Tics Using Intraoral Occlusal Devices: Report of Two Cases

**DOI:** 10.1111/scd.70032

**Published:** 2025-04-14

**Authors:** Andrea Hoette Stahlke, Daniel Bonotto, Danielle Veiga Bonotto, Priscila Brenner Hilgenberg‐Sydney

**Affiliations:** ^1^ Brazilian Dental Association Paraná Division Curitiba Paraná Brazil; ^2^ Federal University of Paraná, Health Sciences Center Department of Restorative Dentistry Curitiba Paraná Brazil

**Keywords:** case report, occlusal splints, tic disorders, Tourette syndrome

## Abstract

**Aims:**

The aim of this study is to present and discuss two cases of patients with Tourette syndrome controlled with intraoral occlusal devices.

**Methods and Results:**

This paper presents two case reports: one of a 10‐year‐old male patient and another of a 17‐year‐old male patient who underwent treatment using a conservative approach that has garnered increasing clinical interest: the use of an occlusal device. This device is placed over the lower teeth, creating a space between the mandible and maxilla. Patients reported improvements in tic frequency as well as enhancements in their ability to focus and concentrate on academic activities following the installation of the occlusal device.

**Conclusions:**

The use of these occlusal devices demonstrates promising success in managing tics associated with Tourette syndrome.

## 1 Introduction

For a century and a half, Tourette syndrome has been a mystery to the medical profession, with professionals believing it to be a psychological disorder, then a brain neurological disorder, and later an infectious disease caused by streptococci. What was never considered was that this disorder was due to a structural deformity that would manifest as a neurological problem. What was discovered is that Tourette syndrome is not of psychological, infectious, genetic, or environmental origin, but according to Sims and Stak [1], it is what is called a structural‐reflex disorder.

Historically, Dr. Gilles de la Tourette published in 1885 the “Study of a Nervous Affliction,” in which he described the syndrome in nine patients [2], which would later give the syndrome its name. Currently, it is classified by the American Psychiatric Association as a mental disorder. It is defined as a chronic, idiopathic, hereditary neurological disorder characterized by the presence of multiple motor tics and at least one vocal tic, which generally begins between the ages of 5 and 8 [3, 4].

Tics can be mild and not bothersome, but they can cause physical discomfort, academic and professional impairment, and social disability. The tics in Tourette syndrome can be associated with various comorbidities, including anxiety, attention deficit hyperactivity disorder (ADHD), misophonia, and obsessive‐compulsive disorder (OCD). Tics can occur at any age in childhood, but generally around 2–18 years, with a peak around 6 years old. The peak severity of tics is often in the second decade, around 10–12 years, and then they decrease or cease completely by late adolescence, although some may persist into late adulthood [5, 6, 7]. When tics impair the patient's quality of life, there are many established treatment options that can be considered: behavioral interventions, intraoral devices, medications, surgical interventions, and neuromodulatory approaches [3, 5].

Although pharmacotherapy plays an important role in the treatment of Tourette syndrome, it is not a cure, and the treatments have side effects and potential risks. The side effects of medications are common and can accumulate over time. Several medications have been prescribed for the treatment of Tourette syndrome. These medications are neuroleptics, which cause significant side effects: fatigue, nausea, dystonia, dysregulation of body temperature, headaches, hallucinations, and cognitive difficulties. For this reason, more conservative approaches are often considered first‐line, including psychoeducation, behavioral interventions, biofeedback, and intraoral devices [5].

The aim of this study is to present and discuss two cases of patients with Tourette syndrome in whom both tics and associated bruxism were controlled using intraoral occlusal devices.

## 2 Methods

Two cases of patients who sought treatment for tics will be presented. These patients sought care from a specialist in TMD and Orofacial Pain at a private clinic in the city of Curitiba, Paraná, Brazil. At the time of treatment, the patients' guardians provided written informed consent for the proposed approach, also authorizing the use of the data for scientific publication. Both patients were referred by a Neurologist with a prior medical diagnosis of Tourette Syndrome, in accordance with the Diagnostic and Statistical Manual of Mental Disorders, Fifth Edition [4].

Both patients received the same treatment plan, as an occlusal device was offered as an option to reduce tics.

## 3 Results

### 3.1 Case Reports

#### 3.1.1 Case 1

Case 1 is a 17‐year‐old male patient, single, leukoderma, presented with Tourette syndrome. The physical tics began at the age of 5, and the vocal tics started at 7 years old. The patient has always refused pharmacological treatment for the syndrome, stating that it impairs his full awareness, negatively affecting his social life. The tics occurred dozens of times a day, mainly consisting of motor tics involving the mandible, without verbalization, only producing lower sounds, especially during moments of concentration or heightened emotional tension. Additionally, the patient reported episodes of awake bruxism, primarily linked to his tics.

On dental examination, the patient showed no signs or symptoms of temporomandibular dysfunction. The periodontal condition was healthy, with no carious lesions present at the permanent dentition. The patient was undergoing orthodontic treatment with another dentist for a Class II malocclusion but was in the final stage of treatment.

**FIGURE 1:**
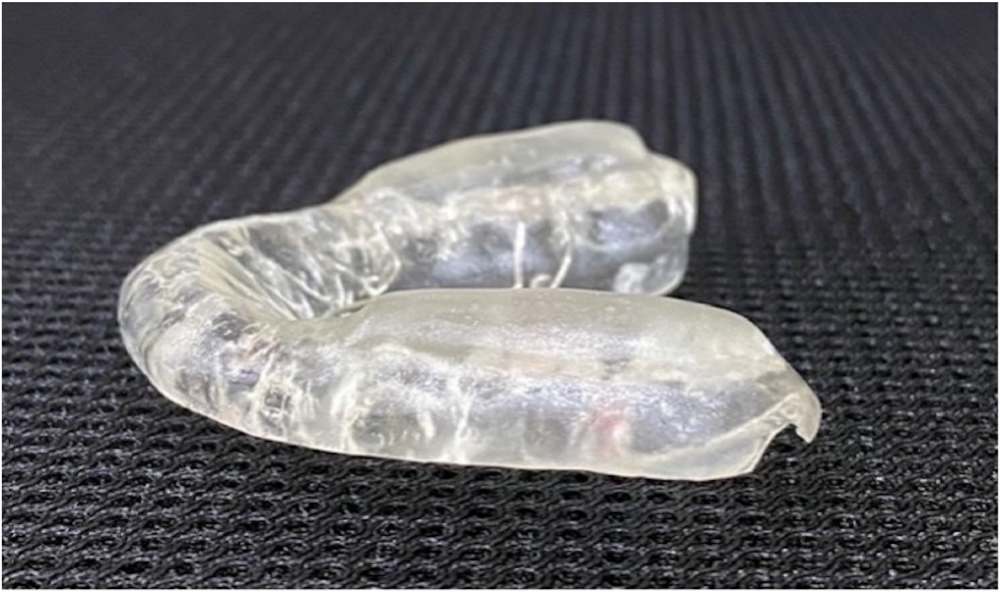
Lower occlusal device, as described by Bennett et al. [8] (Case 1).

To fabricate the occlusal device, the upper and lower arches were molded, and plaster models were created. To determine the necessary vertical jaw height adjustment to observe a decrease in the frequency and/or intensity of the tics, the clinician used tongue depressors, with a maximum of seven, to measure the required adjustment for the participant's tics to subside. The interocclusal record was made at this height, with the tongue depressors placed between the anterior teeth, and dental wax positioned between the upper and lower posterior teeth [8]. During the bite closure, the mandible was gently guided into a comfortable position, ensuring that the participant did not protrude or laterally deviate the jaw while biting the wax, while keeping the tip of the tongue posteriorly on the palate [9]. This interocclusal record was then sent to the dental laboratory to fabricate the occlusal device. This occlusal device was a rigid, transparent, and made of acrylic resin. As this occlusal device is installed in the lower arch and features a posterior lift, allowing dental contact only on the posterior teeth (Figure 1), occlusal adjustment was performed to establish contacts in the intercuspal position, but no disocclusion guides were created [8]. However, the device is not dentate and allows for excentric mandibular movements. This certainly has the potential to cause side effects, such as the extrusion of antagonist teeth over time. However, in Case 1, this was not an initial concern, as the patient was using fixed orthodontic appliances, which could serve as a stabilizing factor for the position of the teeth while the treatment was on standby.

**FIGURE 2:**
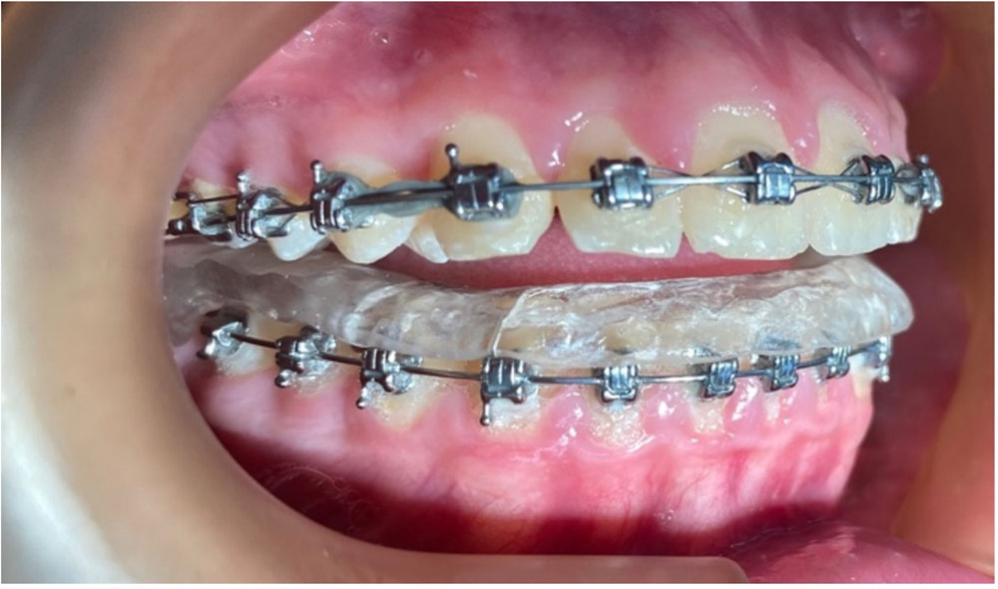
Lower occlusal device in place (Case 1).

There was a slight difficulty in the installation and retention of the occlusal device due to the patient's use of fixed orthodontic appliances (Figure 2). Despite this, the device was successfully placed, and the patient was instructed to wear it for a few hours each day during the initial days to allow for gradual adaptation to its retention.

The patient was instructed to use the occlusal device as needed during waking hours, especially in those moments when tics interfered with concentration, studying or during stressful moments. The occlusal device should not be worn during meals. The patient reported feeling comfortable and experiencing an improvement in tics while the occlusal device was on. A second report was collected a week later when the patient's mother also noticed an improvement in the tics, but especially an improvement in his “focus” during studies. About 15 days after the delivery of the occlusal device, another report was collected from the patient, who noted a slight initial difficulty in adapting to the occlusal device. However, he reported a reduction in the perception of tics, more from his family than from himself. At the same opportunity, the patient reported that the significant gain he noticed during the use of the occlusal device was being able to achieve more focus, attention, and concentration in his academic activities. The patient continues to receive clinical follow‐up and is appropriately informed about the risks and benefits of using this type of occlusal device.

#### 3.1.2 Case 2

Case 2 is a 10‐year‐old male patient diagnosed with Tourette syndrome, with mutilating traits, presented with severe sleep bruxism and high‐intensity wakefulness bruxism events. The stereotyped events characteristic of the syndrome accompanied the episodes of wakefulness bruxism, which were sometimes performed with high levels of force. As prescribed by the neurologist, he was taking 50 mg of sertraline and 2 mg of risperidone daily.

**FIGURE 3:**
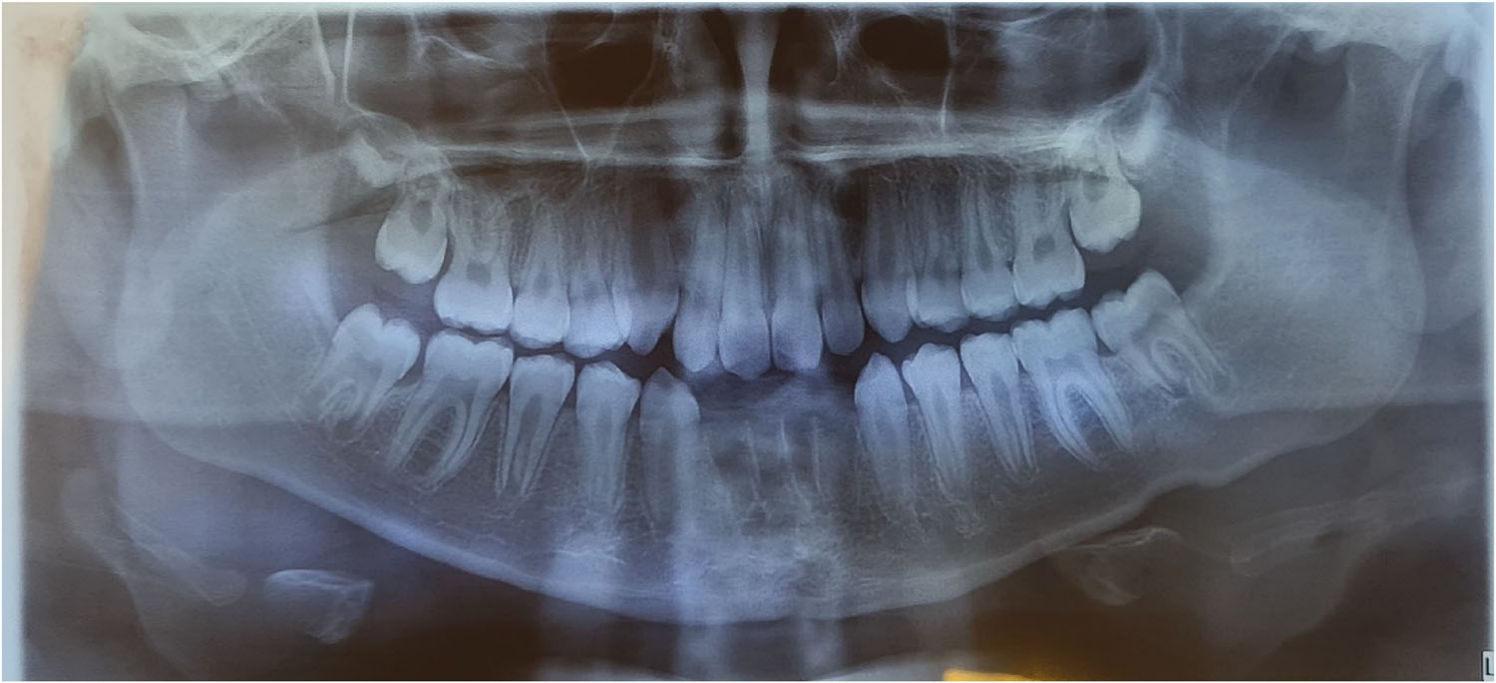
Panoramic radiograph showing recent loss of lower incisors (Case 2).

On dental examination, the patient showed no signs or symptoms of temporomandibular dysfunction. The periodontal condition was healthy, with no carious lesions present. The patient had mixed dentition, a Class I occlusion, anterior open bite and had not undergone previous orthodontic treatment. The patient exhibited significant oral consequences related to the tics and associated bruxism, including tongue and cheek lacerations and loss of the four lower incisors (Figure 3), and signs of occlusal trauma in other lower teeth, with marked mobility.

**FIGURE 4:**
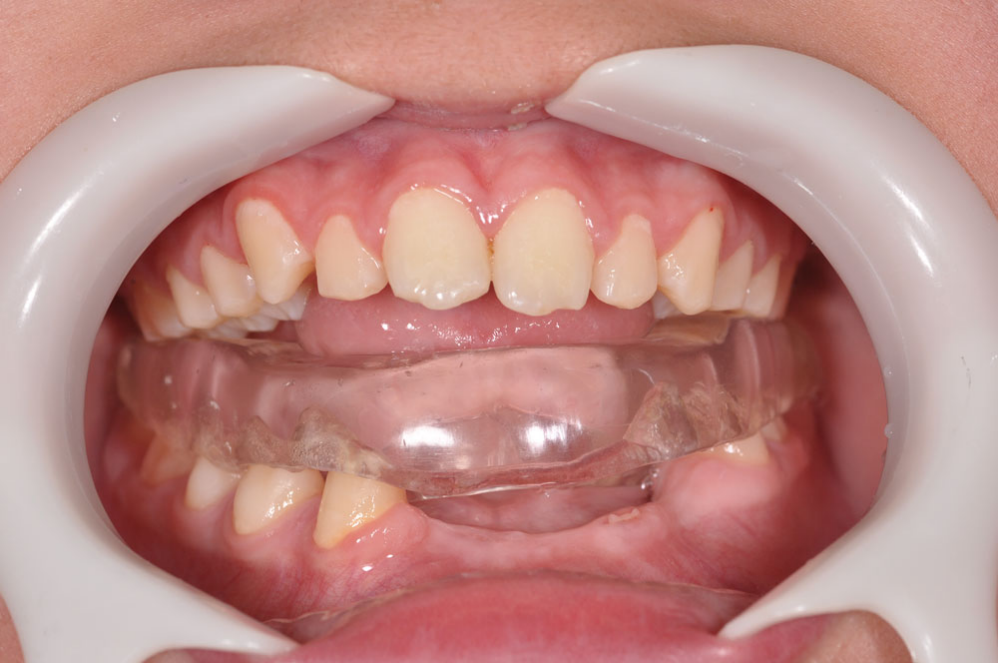
Lower occlusal device in place (Case 2).

Due to these oral consequences and based on Bennett et al. proposals [8], an occlusal device was fabricated as described by these authors (Figure 4), following the same approach previously applied to Case 1. The patient was instructed to use the occlusal device as needed during waking hours, especially in those moments when tics interfered with concentration or during stressful moments. The occlusal device should not be worn during meals. For this patient, if the caregiver noticed episodes of sleep bruxism with teeth grinding noises, they were instructed to have the occlusal device used.

Over the course of 6 months, the patient returned for periodic re‐evaluations to monitor his progress and adjust the occlusal device. As already mentioned, the use of this occlusal device has the potential to cause occlusal changes. This risk was communicated to the caregivers and monitored at each visit. Fortunately, no occlusal changes have been observed during the follow‐up period so far. During this period, he was also referred for a periodontal follow‐up. In the adaptation phase to the occlusal device, he underwent extraction of tooth 33 (see Figure 4).

The proposed approach proved effective in controlling the occlusal pathologies. An improvement was observed in the frequency of motor tics, not only in oral tics but also in head and arm tics. The caregivers also reported a subjective perception of improvement in attention levels in daily activities, positively impacting his academic performance.

## 4 Discussion

The two cases presented demonstrate the relevance of interdisciplinary and alternative approaches in managing patients with Tourette syndrome, especially those with severe oral manifestations such as bruxism and mutilating tics. Both patients, despite differences in age and symptom severity, responded positively to the use of intraoral devices, with improvements in both motor symptoms and their ability to concentrate and perform academically. These case reports highlight the importance of considering conservative and personalized treatments, such as the occlusal device, for controlling tics and occlusal consequences, minimizing the negative impact on these patients' quality of life.

The trigeminal nerve, known to be a tonic regulator of the reticular formation, has a significant influence on the sensorimotor circuits of the brainstem, particularly in controlling posture and locomotion through the descending motor pathways of the brainstem [10]. Given its role in motor regulation, dysfunctions in the central nervous system can trigger involuntary movements of the masticatory muscles, clinically manifesting as bruxism. Bruxism can also be a comorbidity of Tourette syndrome, further highlighting the complex neurological interactions involved in both conditions [11].

The success observed in these two presented cases aligns with other authors who have reported similar experiences in the literature. Sims and Stack, in 2010 [1]; Kwon et al., in 2018 [12]; Murakami et al., in 2019 [13]; and Bennett et al., in 2021 [8] all used an occlusal splint with the aim of controlling Tourette syndrome, and all achieved success in reducing their patients' tics. In the present study, the use of occlusal devices was also for managing the injuries of bruxism. Previous authors have already mentioned the comorbidity of persistent tics and bruxism as comorbid conditions that need to be addressed together [11, 14].

The mechanism by which oral devices seem to work in patients with Tourette syndrome remains a subject of investigation, discussion, and disagreement among authors. The trigeminal nerve is a tonic regulator of the reticular formation, which gives it a strong influence over the brainstem's sensorimotor circuits. Posture and locomotion are controlled by the descending motor pathways from the brainstem's reticular formation. There is a hypothesis that most patients with Tourette syndrome and its various comorbid disorders are affected by an inadequate positioning of the mandible at the base of the skull, resulting in constant stimulation of the trigeminal system below the nociceptive pain threshold. Movement disorders may develop from peripheral nerve injuries in the craniocervical region. In some cases, the auriculotemporal nerve may be compressed, causing neuroinflammation, which could affect the trigeminal ganglion and projections of the auriculotemporal nerve located in the spinal nucleus of the trigeminal nerve and in the brainstem's reticular formation. Neuroinflammation in these posterior regions could act as physiological drivers for abnormal reflexive behaviors and cerebral changes in the nervous system [1, 15, 16].

In 2019, Hottel et al. [16] tested a commercial brand in the United States called Tic Guard. The action of separating the opposing posterior teeth (usually by an excess of 4–5 mm) causes the condyle to rotate within the condylar fossa of the temporomandibular joint (TMJ) and move downward onto the articular eminence of the temporal bone. Opening the bite in this manner creates neurostimulation of the sensory fibers of the joint capsule, which triggers a shutdown mechanism in the brain to prevent motor nerves from firing. The signal received by the brain through the fifth cranial nerve (trigeminal nerve) reduces or eliminates the motor signal from the brain to cranial nerves III, IV, V, VI, VII, IX, and XI. Since common motor tics involve the muscles of the face, neck, and shoulders, and all vocal tics involve oral and pharyngeal structures, this mechanism helps reduce or eliminate tic behaviors.

On the other hand, other authors disagree with the theory of auriculotemporal decompression and propose that the occlusal splint functions as a sensory trick to improve tic symptoms and associated bruxism. According to Murakami et al., the occlusal splint acts as a sensory trick that improves both tic symptoms and dystonia. The efficacy of the sensory trick in dystonia is associated with the neural processing of proprioception. The occlusal splint modulates proprioceptive signals from the jaw‐closing muscle spindles, which are transmitted to the insular cortex. Abnormal insular hyperactivity has also been reported in patients with Tourette syndrome [13].

Tourette syndrome appears to be a highly disabling condition, causing embarrassment and suffering for the patient. Non‐invasive and conservative treatment measures should always be attempted before the use of pharmacological therapies. The case reports presented demonstrate good patient acceptance and a favorable clinical response to the use of an occlusal device for controlling tics associated with Tourette syndrome, as well as managing the injuries of bruxism linked to the condition. The treatment provided valuable insights into the role of occlusal appliances in addressing both tic‐related dental damage and bruxism in Tourette syndrome patients. Patients reported a decrease in tic frequency and intensity, particularly in episodes involving the mandible. Additionally, improved focus and concentration were noted. Moving forward, we recommend further studies with larger sample sizes and a more detailed investigation into the neurological and pathophysiological aspects of Tourette syndrome. Although this condition affects many individuals, there is still a need for stronger scientific evidence regarding dental management approaches.

## 5 Conclusion

The use of these occlusal devices demonstrates promising success in managing tics associated with Tourette syndrome. Both patients and their families showed excellent adherence to the treatment, with high levels of satisfaction regarding the results. The impact of the occlusal device on quality of life exceeded expectations, providing a viable alternative for assisting in the control of tics. However, it is essential to remain mindful of the potential side effects associated with its use, ensuring that the benefits outweigh any risks. Overall, this approach could offer valuable support in the management of Tourette syndrome.

## Conflicts of Interest

The authors declare no conflicts of interest.
